# Disseminated Tuberculosis With Cutaneous, Cerebrovascular, and Thromboembolic Manifestations in a Young Immunocompetent Male Patient: A Case Report

**DOI:** 10.7759/cureus.111589

**Published:** 2026-06-26

**Authors:** Muhammad Usman, Laiba Murtaza, Kiran Paramasivam, Krystal Latchman, Karanpal Dhaliwal

**Affiliations:** 1 Internal Medicine, Mercy Hospital Fort Smith, Fort Smith, USA; 2 Internal Medicine, Arkansas College of Osteopathic Medicine, Fort Smith, USA; 3 Internal Medicine, King Edward Medical University, Lahore, PAK

**Keywords:** cutaneous tuberculosis, disseminated tuberculosis, extrapulmonary manifestations of tuberculosis, immunocompetent adult, infection-induced hypercoagulability, ischemic stroke, mycobacterium tuberculosis complex, venous thromboembolism

## Abstract

Tuberculosis (TB) remains a leading infection because of morbidity and mortality worldwide, with extrapulmonary manifestations occurring less commonly among human immunodeficiency virus (HIV) seronegative individuals. We present the case of a 20-year-old Marshallese male patient with a medical history of hypertension, who presented with dysphagia, weight loss, and altered mental status after a fall and was found to have disseminated TB infection involving the lungs, pleura, and skin of the left auricular region. His hospital course was complicated by a large left middle cerebral artery territory ischemic infarct with mild hemorrhagic transformation and a left lower lobe pulmonary embolism. Of note, the patient was HIV seronegative, and his hypercoagulable workup was also unremarkable, thus suggesting a TB-associated hypercoagulable state as the likely mechanism for his thromboembolic complications. Although markedly elevated pleural fluid adenosine deaminase levels and exudative pleural effusion provided strong corroborating evidence for tuberculous pleuritis, negative *Mycobacterium tuberculosis* complex polymerase chain reaction (PCR) on both bronchoalveolar lavage and serum added to the diagnostic complexity. This case highlights the importance of maintaining a high index of clinical suspicion for disseminated TB and its thromboembolic complications in young patients from high-TB-burden regions, even in the absence of immunosuppression.

## Introduction

Tuberculosis (TB) caused by *Mycobacterium tuberculosis* (MTB) complex remains a global health issue, with approximately 10 million new cases diagnosed annually [1]. Although pulmonary TB is the most common manifestation of this disease, extrapulmonary TB accounts for approximately 20% of cases in HIV seronegative individuals and can affect almost any organ system [2]. 

Disseminated TB is a subtype defined as involvement of two or more distinctly separate anatomic sites and carries significant morbidity and mortality, especially when diagnosis is delayed [2,3]. Cutaneous TB is an uncommon manifestation, comprising only 1-1.5% of all extrapulmonary TB cases [4]. Auricular involvement is particularly rare; a 15-year retrospective analysis of 886 patients of cutaneous TB identified only 20 cases (2.26%) with ear involvement [5]. TB-associated ischemic stroke is a recognized but underreported complication. Although cerebral infarction most commonly occurs in the setting of tuberculous meningitis through mechanisms of vasculitis and arteritis, ischemic stroke has also been described in disseminated TB without central nervous system (CNS) involvement [6]. Population-based studies have demonstrated that survivors of TB have a significantly higher risk of stroke compared with matched controls [7].

TB has also been associated with increased risk of venous thromboembolism (VTE), with the prevalence of VTE among TB patients being approximately 2.07% and an odds ratio (OR) of 1.55 compared with non-TB patients [8]. The pathophysiology involves a systemic hypercoagulable state characterized by high fibrinogen levels, impaired mechanisms of fibrinolysis, decreased levels of protein C and protein S activity, and decreased Antithrombin III levels, leading to widespread thromboembolic complications and active disease [9]. 

The Republic of Marshall Islands is classified among the highest TB burden countries in the Western Pacific region, with an incidence of TB significantly higher when compared to the United States [10]. Here, we present a unique case of disseminated TB in a young, HIV seronegative Marshallese man presenting with cutaneous auricular TB, large territory ischemic stroke, and pulmonary embolism, a triad which has been rarely reported in the literature. 

## Case presentation

A 20-year-old Marshallese male patient with a medical history of hypertension currently under control with low-dose lisinopril (no former etiology known) presented to the hospital with a nosebleed and confusion after a fall. His family reported progressively worsening dysphagia, recurrent febrile episodes, and unintentional weight loss of approximately 20 pounds over the preceding months. A skin lesion behind his left ear was also noted, which had been reportedly present for two months, for which the patient was taking doxycycline without improvement. Due to financial constraints, the patient had difficulty accessing medical care during this time. No prior records regarding the duration or severity of hypertension were available.

On initial evaluation, the patient remained significantly confused. His vital signs were stable except for a fever of 101.2 °F (38.4 °C). Physical examination was significant for a skin lesion in the left auricular area (Figure 1), decreased breath sounds in the right lower lung field with rhonchi in the right upper lung field, and somnolence with withdrawal to painful stimuli.

**Figure 1:**
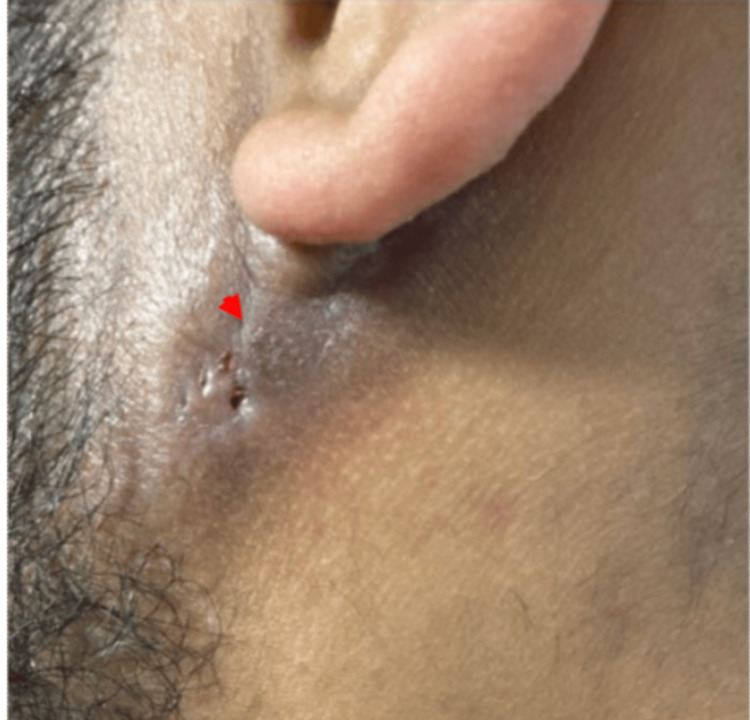
Left postauricular area demonstrating an erythematous, indurated plaque with overlying skin changes, and a central draining sinus tract (arrowhead).

Neurological examination revealed limited attention, and the patient needed repeated direction for following simple motor commands. Cranial nerve examination demonstrated equal and reactive bilateral pupils, visual fields full to confrontation with intact extraocular movements, normal funduscopic examination, no facial asymmetry, tongue midline, hearing intact, limited speech output but articulate without dysarthria, and sensations intact in V1-V3 bilaterally. Motor examination revealed asymmetrical motor weakness with reduced tone in the right upper and lower extremities compared to the left, with pronator drift (3/5 in left extremities, 2/5 in right upper and lower extremities). Muscle bulk was normal throughout. Bilateral upper and lower extremity reflexes were normal with bilateral flexor plantar responses. Sensory examination was also normal. Gait and coordination could not be assessed due to the patient's clinical condition.

Meningeal signs were absent. The patient had no history of diabetes mellitus, malignancy, organ transplantation, chronic renal or hepatic disease, use of immunosuppressive medications including corticosteroids or biologic agents, or known primary immunodeficiency. HIV-1/2 antigen/antibody screening with HIV-1 p24 antigen was nonreactive, the acute hepatitis panel was negative, and the complete blood count showed no cytopenias or findings suggestive of hematological malignancy. Initial lab work was significant for hypokalemia and hypoalbuminemia (Table 1).

**Table 1:** Results of pertinent laboratory tests at admission

Parameter	Patient Value	Reference Range
White Blood Cell Count	5.1 K/uL	4.2-9.1 K/uL
Red Blood Cell Count	4.00 M/uL	4.63-6.08 M/uL
Hemoglobin	11.2 g/dL	13.7-17.5 g/dL
Hematocrit	33.4 %	40.1-51.0 %
Platelet Count	405 K/uL	163-337 K/uL
Neutrophils	69 %	34-71 %
Lymphocytes	15 %	19-53 %
Monocytes	8 %	5-13 %
Eosinophils	7 %	1-7 %
Basophils	0 %	0-1 %
Immature Granulocytes	1 %	0-1 %
Sodium	135 mmol/L	136-145 mmol/L
Potassium	3.2 mmol/L	3.5-5.1 mmol/L
Chloride	102 mmol/L	98-107 mmol/L
Carbon Dioxide	22 mmol/L	22-29 mmol/L
Calcium	8.2 mg/dL	8.6-10.0 mg/dL
Blood Urea Nitrogen	9 mg/dL	6-20 mg/dL
Creatinine	1.00 mg/dL	0.67-1.17 mg/dL
Glucose	97 mg/dL	74-99 mg/dL
Total Protein	6.9 g/dL	6.4-8.3 g/dL
Albumin	2.0 g/dL	4.0-4.9 g/dL
Total Bilirubin	<0.2 mg/dL	0.0-1.2 mg/dL
Alkaline Phosphatase	116 U/L	40-129 U/L
Aspartate Aminotransferase	21 U/L	0-40 U/L
Alanine Aminotransferase	12 U/L	≤41 U/L
Magnesium	1.8 mg/dL	1.6-2.6 mg/dL
Pro B-Type Natriuretic Peptide	120 pg/mL	≤ 125 pg/mL
Procalcitonin	0.45 ng/mL	≤ 0.08 ng/mL
Thyroid-Stimulating Hormone	2.47 uIU/mL	0.27-4.20 uIU/mL
Creatine Kinase	40 U/L	22-200 U/L
Total Cholesterol	225 mg/dL	< 200 mg/dL
Triglycerides	122 mg/dL	< 150 mg/dL
Low-Density Lipoprotein Cholesterol	169 mg/dL	< 100 mg/dL
Urine Drug Screen	Unremarkable	Unremarkable

Chest radiograph revealed pulmonary infiltrates in the right mid and lower lung zones with moderate right-sided pleural effusions (Figure 2). A contrast-enhanced computed tomography (CT) of the chest confirmed these findings and additionally identified a left lower lobe pulmonary embolism for which heparin infusion was initiated. A follow-up transthoracic echocardiogram was negative for any right heart strain or patent foramen ovale. 

**Figure 2:**
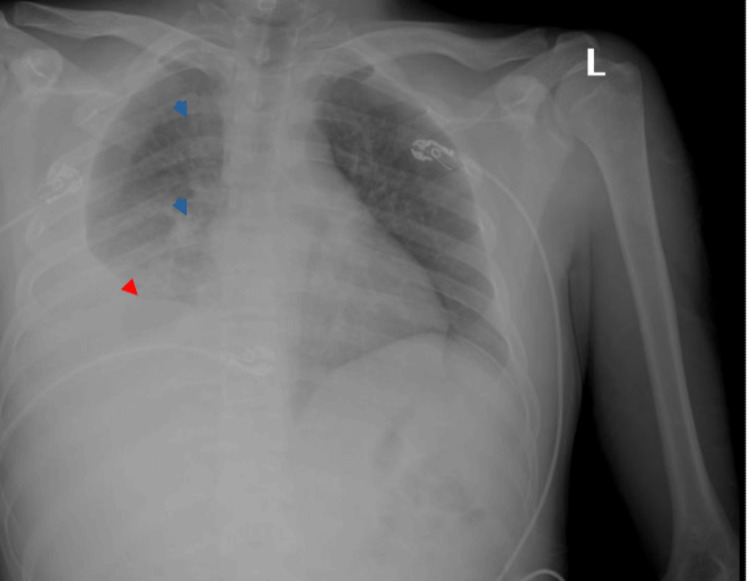
Chest radiograph demonstrating heterogeneous opacification in the right lower lung zones consistent with pulmonary infiltrates and a moderate right-sided pleural effusion with blunting of the right costophrenic angle (red arrowhead). Patchy airspace opacities are also noted in the right upper and middle lung zones (blue arrowheads).

Due to the patient's ongoing confusion, a CT of the head without contrast was performed, which revealed a large region of hypodensity with loss of gray-white matter differentiation in the left cerebral hemisphere, raising suspicion for acute ischemia. A CT angiogram of the head and neck demonstrated large, ill-defined hypodensity in the left frontal, temporal, and parietal lobes without any major intracranial aneurysm, stenosis, or occlusion, and patent intracranial venous sinuses. Heparin infusion was immediately held due to concern for hemorrhagic transformation. 

Magnetic resonance imaging (MRI) of the brain with and without contrast revealed an 11.5 cm x 4 cm acute ischemic infarct with mild hemorrhagic transformation in the left frontal, temporal, and parietal lobes in the left middle cerebral artery (MCA) vascular distribution, with the likely mechanism of reperfusion injury for hemorrhagic transformation (Figure 3).

**Figure 3:**
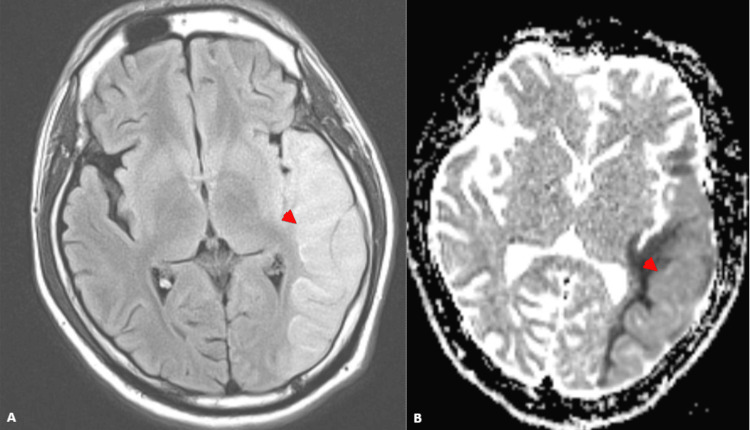
MRI of the brain demonstrating large territory left middle cerebral artery ischemic infarct. (A) Axial T2 motion-corrected sequence showing extensive hyperintense signal involving the left frontal, temporal, and parietal lobes in the left middle cerebral artery (MCA) vascular distribution, consistent with acute ischemic infarction (arrowhead). (B) Axial diffusion-weighted imaging (DWI) sequence demonstrating corresponding restricted diffusion within the left MCA territory (arrowhead), confirming acute ischemia.

An electroencephalogram (EEG) was also done, which was negative for any epileptiform changes or electrographic seizures. A lumbar puncture was not performed due to negative physical exam findings. 

Due to the concerns of underlying infectious processes, infectious disease and pulmonology were consulted. Bronchoscopy with bronchoalveolar lavage (BAL) and thoracentesis with pleural fluid analysis were performed. BAL analysis, pleural fluid analysis, and further infectious workup are summarized in Table 2.

**Table 2:** Infectious workup HBsAg: hepatitis B surface antigen; IgM: immunoglobulin M; HAV: hepatitis A virus; HCV: hepatitis C virus; BAL: bronchoalveolar lavage

Test Name	Test Value	Reference Range
Blood Cultures	No growth	No growth
Human Immunodeficiency Virus 1 and 2 Antibodies with HIV-1 Antigen Screen	Nonreactive	Nonreactive
Acute Hepatitis Panel (HBsAg, Core IgM, HAV IgM, HCV Antibody)	Negative	Negative
Pneumonia Pathogen Polymerase Chain Reaction Panel	Negative for nucleic acids	Negative
Left Auricular Lesion Drainage Acid-Fast Bacilli Culture	Positive for *Mycobacterium tuberculosis* complex	Negative
BAL: Neutrophils	40 %	0-1 %
BAL: Lymphocytes	60 %	18-36 %
BAL: Total Nucleated Cells	152 /uL	1395-3734 /uL
BAL: Fungal Cultures	Negative	Negative
BAL: Respiratory Cultures with Gram Stain	Negative	Negative
BAL: Acid-Fast Bacilli Culture	Positive for *Mycobacterium tuberculosis* complex	Negative
BAL: Mycobacterium tuberculosis complex Polymerase Chain Reaction	Negative	Negative
Lactate Dehydrogenase Levels (Pleural Fluid)	319 U/L	< 200 U/L (Transudate) / > 200 U/L (Exudate)
Pleural Fluid Protein	3.5 g/dL	< 2 g/dL (Transudate) / > 2.0 g/dL (Exudate)
Pleural Fluid Adenosine Deaminase	102.4 U/L	< 9.2 U/L
Serum Mycobacterium tuberculosis complex Polymerase Chain Reaction	Negative	Negative
Serum Beta-D-Glucan Assay	Less than 31 pg/mL	< 60 pg/mL
Serum Aspergillus Galactomannan Antigen	Less than 0.50	< 0.50
Serum Cryptococcal Antigen	Negative	Negative
Urine Histoplasma Antigen	Less than 0.2 ng/mL	< 0.2 ng/mL
Urine Blastomyces Antigen	Negative	0.31-20 ng/mL
Rapid Plasma Reagin	Nonreactive	Non-reactive
Treponema Pallidum Particle Agglutination Assay	Nonreactive	Non-reactive

TB was ultimately confirmed by positive mycobacterial cultures from BAL and the retroauricular lesion despite negative MTB PCR testing. The patient was placed on airborne precautions and was started on rifampin, isoniazid, pyrazinamide, and ethambutol (RIPE) by the infectious disease team with close monitoring of liver transaminases. His mentation improved with anti-TB therapy, resolution of febrile illness, and appropriate ischemic stroke management. The patient was also ultimately started on heparin infusion for pulmonary embolism after stable findings of ischemic infarct on follow-up imaging after multidisciplinary discussion. Due to embolic mechanisms of stroke and pulmonary embolism, hematology was also consulted for a hypercoagulable workup, summarized in Table 3.

**Table 3:** Hypercoagulable workup. APTT: activated partial thromboplastin time; INR: international normalized ratio

Test Name	Test Value	Reference Range
Cardiolipin IgG Antibody	< 2.0 U/mL	< 20 U/mL
Cardiolipin IgM Antibody	< 2.0 U/mL	< 20 U/mL
Cardiolipin IgA Antibody	2.3 U/mL	< 20 U/mL
Beta-2 Glycoprotein I IgG Antibodies	< 2.0 U/mL	< 20 U/mL
Beta-2 Glycoprotein I IgM Antibody	< 2.0 U/mL	< 20 U/mL
Beta-2 Glycoprotein I IgA	2.9 U/mL	< 20 U/mL
Lupus Anticoagulant Assay	Negative	Negative
Prothrombin Factor II Mutation Analysis	Negative	Negative
Factor V Leiden Mutation	Negative	Negative
Protein C Activity	104%	70% – 141%
Protein S Activity	56%	45% – 125%
Antithrombin III Activity	121%	82% – 122%
Pro Time (Prothrombin Time)	12.6 seconds	11.8 – 14.8 seconds
APTT	33.6 seconds	22.7 – 36.7 seconds
INR	0.9	0.9 – 1.2

Repeat contrast-enhanced CT angiogram of the chest was negative for any pulmonary embolism. With a negative hypercoagulable workup and no pulmonary embolism on repeat imaging, systemic anticoagulation was discontinued after multidisciplinary discussion. The patient was started on dual antiplatelet therapy (DAPT) for ischemic stroke management. The patient's overall clinical condition improved significantly on RIPE therapy. He was established with the state health department for continuation of his TB treatment with RIPE therapy. 

At the two-month follow-up, the patient denied having any symptoms and was continuing his RIPE therapy at the state health department without any issues. A brief summary of the case is shown in Figure 4.

**Figure 4:**
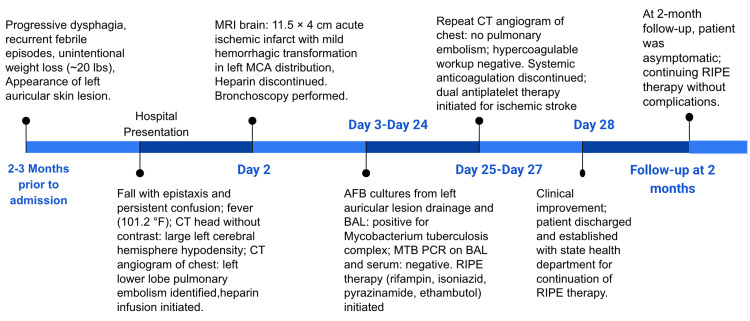
Clinical timeline summarizing the chronological sequence of presenting symptoms, diagnostic evaluations, and therapeutic interventions during the hospital course and follow-up. AFB: acid-fast bacilli; BAL: bronchoalveolar lavage; CT: computed tomography; MCA: middle cerebral artery; MRI: magnetic resonance imaging; MTB: *Mycobacterium tuberculosis*; PCR: polymerase chain reaction; RIPE: rifampin, isoniazid, pyrazinamide, ethambutol

## Discussion

This case highlights several unique and clinically significant aspects of disseminated TB. The simultaneous presentation of cutaneous TB involving the auricular area, large territory MCA ischemic stroke, and pulmonary embolism in a young HIV-negative, immunocompetent patient presents an exceedingly rare constellation of symptoms. Cutaneous TB of the auricular area is itself exceptionally uncommon [5]. In our patient, the post-auricular lesion was misdiagnosed and treated with doxycycline without any improvement, highlighting the diagnostic challenge posed by cutaneous TB, which can mimic chronic eczematous processes or other cutaneous infectious etiologies [4,5]. 

Also, the occurrence of a large territory ischemic stroke in the MCA vascular distribution in our patient case is of utmost significance. Whereas TB-associated cerebral infarction most commonly occurs in the context of tuberculous meningitis secondary to leptomeningeal exudates causing endarteritis and vasculitis, ischemic stroke has also been described in disseminated TB without tuberculous meningitis [6]. The absence of meningeal signs on physical examination, normal CT angiography, and patent venous sinuses in our patient suggested that the ischemic stroke was not caused by TB endarteritis or vasculitis. It, rather, favored a TB-induced hypercoagulable state, likely playing a central role in thromboembolic manifestations by inducing a procoagulant state and reducing natural anticoagulants, including protein C, protein S, and antithrombin III [9]. Our patient's hypercoagulable workup was negative for any acquired or inherited thrombophilia, further supporting TB-induced hypercoagulability as the mechanism rather than an underlying thrombophilia disorder.

The concurrent pulmonary embolism adds to the complexity of our case. Active TB has been identified as a significant risk factor for VTE, with an OR of 1.55, which is comparable to the risk associated with malignancy [8]. This systemic net procoagulant state secondary to active pulmonary TB appears to be the predominant mechanism for VTE, rather than local intrathoracic venous compression [8,9]. 

In our case, management was further complicated by the need for systemic anticoagulation for pulmonary embolism in the setting of hemorrhagic transformation of the ischemic infarct. After multidisciplinary discussion, anticoagulation was initially held and resumed only after stable findings on follow-up neuroimaging. With resolution of pulmonary embolism on repeat imaging and a negative hypercoagulable workup, anticoagulation was discontinued, and the patient was transitioned to dual antiplatelet therapy for secondary stroke prevention. This clinical scenario underscores the importance of a multidisciplinary approach when managing competing cerebrovascular and thromboembolic complications of disseminated TB.

Of note, this case highlights an important diagnostic discordance. The MTB complex PCR was negative on both BAL and serum despite positive acid-fast bacilli cultures and elevated ADA levels. This discordance is well recognized; the American Thoracic Society (ATS)/Infectious Disease Society of America (IDSA)/Centers for Disease Control and Prevention (CDC) guidelines note that the nucleic acid amplification testing (NAAT) has a sensitivity of only 56%, indicating a false-negative rate of 44%, while specificity remains high at 98% [3]. The guidelines also emphasized that negative PCR testing may not be used to exclude extrapulmonary TB because of extremely common false-negative results. 

TB cultures remain the gold standard with superior sensitivity but are often time-consuming and require weeks for results. In contrast, the significantly elevated pleural fluid ADA levels provided rapid and compelling supportive evidence for tuberculous pleuritis in our case. The ATS/IDSA/CDC guidelines also report that the ADA levels in pleural fluid have a sensitivity of 89-99% and specificity of 88-97% for tuberculous pleural effusion [3]. The degree of elevation of ADA levels in our case is highly characteristic of TB. Our case demonstrates the complementary roles of ADA and cultures in the diagnostic workup of suspected TB, particularly when molecular testing yields false-negative results. 

Finally, this case highlights the impact of social determinants of health on TB diagnosis and outcomes. Our patient was originally from the Republic of the Marshall Islands, which is a high TB burden area in the Western Pacific region [10]. Financial constraints and limited access to healthcare contributed to significant diagnostic delay, allowing disease dissemination and the development of life-threatening complications. This case reinforces the need for increased clinical awareness, targeted public health interventions, and improved access to care in high-risk populations.

## Conclusions

This case demonstrates the ability of disseminated TB to present with a rare and life-threatening combination of cutaneous involvement of the auricular area, large territory ischemic stroke, and pulmonary embolism in a young immunocompetent patient. The TB-associated hypercoagulable state is an underrecognized phenomenon for thromboembolic complications that can occur even in the absence of tuberculous meningitis or identifiable thrombophilia. The markedly elevated pleural fluid ADA levels provided critical early diagnostic support in the setting of false-negative PCR results, thus reinforcing the value of a multimodal diagnostic approach. This case also highlights the importance of maintaining a high index of clinical suspicion for TB and its systemic complications in patients from high-risk regions. Early diagnosis and appropriate initiation of antitubercular therapy, combined with appropriate management of thromboembolic complications, can lead to favorable outcomes as demonstrated by our case. 
